# A novel patient-derived intra-femoral xenograft model of bone metastatic prostate cancer that recapitulates mixed osteolytic and osteoblastic lesions

**DOI:** 10.1186/1479-5876-9-185

**Published:** 2011-10-28

**Authors:** Omer Raheem, Anna A Kulidjian, Christina Wu, Young B Jeong, Tomonori Yamaguchi, Kristen M Smith, Daniel Goff, Heather Leu, Sheldon R Morris, Nicholas A Cacalano, Koichi Masuda, Catriona HM Jamieson, Christopher J Kane, Christina AM Jamieson

**Affiliations:** 1 Moores Cancer Center, University of California, San Diego, La Jolla, CA, USA; 2 Division of Urology, Department of Surgery, UCSD, La Jolla, CA, USA; 3 Division of Orthopedic Surgery, Dept. of Surgery, UCSD, La Jolla, CA, USA; 4 Dept. of Medicine, UCSD, La Jolla, CA, USA; 5 Dept. of Radiation Oncology, UCLA, Los Angeles, CA, USA; 6 Department of Urology, Chonbuk National University, Jeonju, South Korea

## Abstract

Prostate cancer metastasizes to bone in the majority of patients with advanced disease leading to painfully debilitating fractures, spinal compression and rapid decline. In addition, prostate cancer bone metastases often become resistant to standard therapies including androgen deprivation, radiation and chemotherapy. There are currently few models to elucidate mechanisms of interaction between the bone microenvironment and prostate cancer. It is, thus, essential to develop new patient-derived, orthotopic models. Here we report the development and characterization of PCSD1 (Prostate Cancer San Diego 1), a novel patient-derived intra-femoral xenograft model of prostate bone metastatic cancer that recapitulates mixed osteolytic and osteoblastic lesions.

**Methods:**

A femoral bone metastasis of prostate cancer was removed during hemiarthroplasty and transplanted into *Rag2^-/-^;γ_c_^-/- ^*mice either intra-femorally or sub-cutaneously. Xenograft tumors that developed were analyzed for prostate cancer biomarker expression using RT-PCR and immunohistochemistry. Osteoblastic, osteolytic and mixed lesion formation was measured using micro-computed tomography (microCT).

**Results:**

PCSD1 cells isolated directly from the patient formed tumors in all mice that were transplanted intra-femorally or sub-cutaneously into *Rag2^-/-^;γ_c_^-/- ^*mice. Xenograft tumors expressed human prostate specific antigen (PSA) in RT-PCR and immunohistochemical analyses. PCSD1 tumors also expressed AR, NKX3.1, Keratins 8 and 18, and AMACR. Histologic and microCT analyses revealed that intra-femoral PCSD1 xenograft tumors formed mixed osteolytic and osteoblastic lesions. PCSD1 tumors have been serially passaged in mice as xenografts intra-femorally or sub-cutaneously as well as grown in culture.

**Conclusions:**

PCSD1 xenografts tumors were characterized as advanced, luminal epithelial prostate cancer from a bone metastasis using RT-PCR and immunohistochemical biomarker analyses. PCSD1 intra-femoral xenografts formed mixed osteoblastic/osteolytic lesions that closely resembled the bone lesions in the patient. PCSD1 is a new primary prostate cancer bone metastasis-derived xenograft model to study metastatic disease in the bone and to develop novel therapies for inhibiting prostate cancer growth in the bone-niche.

## Background

Prostate cancer metastasis to bone leads to debilitating fractures and severe bone pain in men with advanced disease for which there is no treatment and is associated with poor prognosis and rapid decline [1]. Recent studies have shown that 100% of men who die of prostate cancer have bone metastases [2]. Paget's "seed and soil" hypothesis posits that the affinity that certain cancers have for bone may be due to a supportive microenvironment for tumor growth [3,4].

Androgen ablation therapy is standard-of-care for advanced prostate cancer, however, bone metastatic prostate cancer often becomes castration-resistant [5]. Two treatments that target the bone microenvironment - bisphosphonates, eg. zolendronic acid, and the RANKL inhibitor, denosumab, which inhibit osteoclasts and osteolysis - have been effective in delaying the onset of skeletal related events (SREs) and new bone metastases in bone metastatic cancers with primarily osteolytic bone lesions [6-11]. A characteristic of prostate cancer bone metastases, however, is that they typically produce osteoblastic or mixed osteoblastic/osteolytic bone lesions that are not as efficiently treated with the osteoclast inhibitors [12-16]. There is currently no curative treatment for prostate cancer bone metastases [1].

A major limitation in understanding and treating prostate cancer bone metastatic disease is that primary human prostate cancer bone metastasis tissues are rarely available for direct analysis or for the development of predictive model systems [1,2]. In addition, spontaneous bone metastasis of prostate cancer is a rare event in murine models of prostate cancer [4,17-19]. Direct injection of prostate cancer cells into the endosteal space of murine leg bones has, thus, provided a robust and reproducible method for studying the growth of prostate cancer in the bone-niche [20]. Prostate cancer cell lines such as LAPC4, LNCaP, LuCAP23.1 and LuCAP35.1 - all of which originated from lymph node metastases - were directly injected into bone either intra-femorally or intra-tibially where they formed tumors and induced bone lesions [4,20,21]. However, direct injection models into the bone-niche using prostate cancer cell lines that did not originate from patient bone metastases may not reflect physiological interactions. The C4-2B cell line is an improvement in this respect since it arose from the sub-cutaneously xenografted LNCaP tumor that spontaneously metastasized to bone within a SCID mouse and formed mixed osteoblastic/osteolytic lesions [22,23]. An intriguing alternative prostate cancer xenograft model assessed metastasis to adult human bone implanted in the hindlimbs of SCID mice [24]. Prostate cancer cells from xenograft tumors homed to the human bone and induced osteolytic lesions but only at low frequencies [24].

Direct bone-injection murine xenograft models using patient-derived bone metastatic prostate cancers, on the other hand, are both an orthotopic and highly tractable xenograft model system [4,20,25-28]. In patients in whom the bone metastatic tumor is causing pathologic fractures, orthopedic surgery is performed to stabilize the bone and primary prostate cancer bone metastases may be collected for study at this time [29,30]. Currently, there are three prostate cancer bone metastasis-derived orthotopic bone xenograft models, PC3, LAPC9 and VCaP [31,32]. Xenograft transplantation of these cell lines into bone demonstrated the range of bone lesions produced by prostate cancer bone metastases: PC3 formed purely osteolytic lesions in intra-tibial xenografts [25,27,28,31], VCaP produced mixed osteoblastic/osteolytic lesions [32], while LAPC9 formed purely osteoblastic lesions [25-28,31].

These models have led to important insights, however, it is crucial to expand on the limited number of existing prostate cancer bone metastasis-derived models in order to understand variability between different patient-derived tumors [21,33,34]. Next-generation genomic DNA sequencing and RNASeq profiling of expression and splice isoforms have revealed significant molecular diversity and complexity of prostate cancers [35-37]. In addition, the existing cell lines have been passaged *ex vivo *for over a decade which has led to progressive alteration of the cell lines away from the original patient characteristics [21,22]. LAPC9 xenografts, for example, generated androgen-independent derivatives that progressed in castrated SCID mice after passaging in mice [33,34]. Genome-wide expression and integrative genomic profiling have comprehensively shown that there are differences between cell lines in vitro compared with primary patient tumors [35-37]. Genome-wide analysis of DNA methylation patterns comparing normal prostate tissue to primary prostate cancer and cell lines revealed a complex picture with some methylation patterns consistently retained in prostate cancer tumors and cell lines while others were distinct [38]. It is essential, therefore, to develop new patient-derived, orthotopic bone metastasis prostate cancer xenograft models that are closer to patients' original tumors especially for determining predictive therapy response profiles [39-42]. In this report we describe the development and characterization of a new patient-derived bone metastatic prostate cancer femoral injection murine model, PCSD1.

## Methods

### Tumor xenograft preparation

A primary prostate cancer bone metastasis sample was obtained from a lytic lesion in the proximal femur from a patient with castrate-resistant prostate cancer with mixed osteoblastic and osteolytic bone metastases and a Gleason score of 9 (5+4). Tumor specimen was prepared aseptically in biohazard safety cabinet according to standard protocols with minor modifications [28,43-47]. Specimen was first minced with sterile razor blades to 1-3 mm^3 ^sized pieces. A portion of the minced tumor was snap frozen for genomic DNA and RNA extraction, cryopreserved in 10%DMSO/90% FBS, or fixed for immunohistochemistry. For sub-cutaneous transplantation, the minced tumor was mixed 1:1 with High Concentration Matrigel (Becton-Dickinson) and 0.1 ml was injected. For intra-femoral injection, the minced tumor sample was disaggregated by digestion in Accumax (Millipore), filtered through sterile, mesh filter (Falcon). Dissociated cells were centrifuged at 1200 RPM, 5 minutes, 4°C, washed three times and resuspended in Iscove's modified DMEM media, 10% FBS at 6.7 × 10^6 ^cells/ml. Cells were mixed 1:1 with high concentration Matrigel for intra-femoral injection of 50,000 cells in 15 μl. Remainder of the dissociated tumor cells were cryopreserved or used for DNA and RNA purification. All studies with human subjects were conducted with the approval of the University of California, San Diego School of Medicine Institutional Review Board. All patients provided written informed consent.

### Surgical technique

All animal protocols were preformed under a UCSD animal welfare IACUC approved protocol. Sub-cutaneous injections were performed using standard protocols [26,28,33]. Briefly, male *Rag2^-/-^;γ_c_^-/- ^*mice 6-8 weeks old were anesthetized with ketamine/xylamine, skin sterilized with 70% ethanol, a 2-3 mm incision was made with autoclaved dissection scissors, a trochar (10 ml *LDEV-Free, 14-gauge catheter (SC injections) Terumo 14 G IV Catheter) was used to inject 100 ul of tumor/matrigel mix below skin right flank, skin flaps were brought together and sealed with VetBond, mice revived post-surgery with Antisedan injected sub-cutaneously at base of neck ruff. For intra-femoral injections mice were anesthesized by intra-peritoneal injection of a mix of 100 mg/kg ketamine and 10 mg/kg Xylazine and injections performed in a BL2 biosafety cabinet. Right hind limb was prepared under standard sterile conditions with 70% ethanol. Knee was held in flexed position and 25 G needle (Monoject 200 25 × 5/8A) was used to make a port in the femoral plateau until there was no resistance that was used as a guide-hole for injection of 15 ul of the tumor cell/Matrigel suspension using a 0.3 ml syringe and 27 G needle. Injection of sample was performed slowly with minimal resistance. Needle was withdrawn and leg immediately straightened, dabbed with antibiotic ointment (RX Neomycin, Polymyxin, Bacitracin Ophthalmic Ointment USP Sterile NDC 13985-017-55), on a sterile cotton tipped applicator (Q-Tips) and held for straight for approximately 1 minute. Mice were injected with Antisedan and placed on a warm Deltaphase Isothermal Pad, and carefully watched during recovery until ambulatory and active.

### Cells and Reagents

Prostate cancer cell lines: LAPC4, was a gift from Dr. Lily Wu, UCLA, and VCaP, purchased from ATCC, were maintained in Iscove's media, 10%FBS, penicillin-streptomycin and K562, a chronic myelogenous leukemia cell line in 10% heat-inactivated FBS, RPMI, Pen-strep.

### RT-PCR

Genomic DNA and RNA were extracted using mortar and pestle pulverization of flash frozen tumor pieces in liquid nitrogen and the Qiagen All-prep kit [47]. RNA was re-purified with RNeasy and treated with RNAse-free, DNase to remove contaminating genomic DNA. For cell lines and purified, dissociated xenograft tumor cells, RNA was extracted using Qiagen RNeasy mini-prep kit. cDNA synthesis was performed with Superscript III (Invitrogen, Inc.) according to manufacturer's protocol, and used for PCR (Taq polymerase, Monserate Biotechnology Group LLC, San Diego, CA). RT-PCR products were resolved on 1% agarose gels. All RT-PCR primers are shown in Table 1. RT-PCR products of the correct size were verified by sequencing (Retrogen, Inc., San Diego, CA).

**Table 1:** Primer sequences for RT-PCR analysis.

Oligo Name*	Oligo Sequence (5' -- 3')*
h-PSA-F	ACCATGTGGGTCCCGGTTGT
h-PSA-R	GAGTTGATAGGGGTGCTCAGG
h-ARfl-F [66,67]	ACATCAAGGAACTCGATCGTATCATTGC
h-ARfl-R [66,67]	TTGGGCACTTGCACAGAGAT
h-ARv567es-F [66,67]	CCAAGGCCTTGCCTGATTGC
h-ARv567es-R [66,67]	TTGGGCACTTGCACAGAGAT
h-KRT5-F2	CACCAAGACTGTGAGGCAGA
h-KRT5-R2	CCTTGTTCATGTAGGCAGCA
h-KRT8-F [68]	CCTCATCAAGAAGGATGTGGA
h-KRT8-R [68]	CACCACAGATGTGTCCGAGA
h-KRT14-F [68]	GACCATTGAGGACCTGAGGA
h-KRT14-R [68]	ATTGATGTCGGCTTCCACAC
h-KRT18-F1	CCAGTCTGTGGAGAACGACA
h-KRT18-R1	CTGAGATTTGGGGGCATCTA
h-KRT18-F2	CCAGTCTGTGGAGAACGACA
h-KRT18-R2	ATCTGGGCTTGTAGGCCTTT
h-NKX3.1-F [46]	GGCCTGGGAGTCTTTGACTCCACTAC
h-NKX3.1-R [46]	ATGTGGAGCCCAAACCACAGAAAATG
h-AMACR-F [69]	CGCGGTGTCATGGAGAAACT
h-AMACR-R [69]	CTTCCTGACTGGCCAAATCC
h-GAPDH-F [70]	GGTGGTCTCCTCTGACTTCAACA
h-GAPDH-R [70]	TTGCTGTAGCCAAATTCGTTGT
m-Gapdh-F [71]	TGTTCCTACCCCCAATGTGT
m-Gapdh-R [71]	GGTCCTCAGTGTAGCCCAAG
TMPRSS2-ERG-F[72]	TAGGCGCGAGCTAAGCAGGAG
TMPRSS2-ERG-R[72]	GTAGGCACACTCAAACAACGACTGG

### Immunohistochemistry

PSA immunostaining was carried out using rabbit anti-human PSA antibody (DAKO A0562) using standard protocols [48] performed by the Moores Cancer Center Histology Core, UCSD, La Jolla, CA. Paraformaldehyde-fixed and paraffin embedded sections from sub-cutaneous PCSD1 xenografts were mounted and 6-μm sections were stained with H & E, anti-PSA, or rabbit IgG isotype control (DAKO N1699) using HRP goat anti-Rabbit as secondary antibody (Jackson 111-035-144) and AEC (Vector SK4200). For intra-femoral tumors, the tumor plus femur and tibia were dissected out as one, formalin-fixed and EDTA de-calcified according to Lavoie et al. [49]. Tissues were mounted in OCT, 6 μm cryosections were fixed in acetone, blocked in 1%BSA/PBS, incubated with anti-PSA or IgG isotype control antibody then processed as above.

### Micro CT Analyses

Femurs of mice injected intra-femorally with PCSD1 were scanned by micro-computed tomography (μCT) SkyScan 1076 (Skyscan, Belgium) at the maximal potential 60 kV and 167 μA with 0.5 mm thick aluminum filter and at the voxel resolution of 9 μm. The μCT scans were performed over 360° of total rotation with each angular rotation step of 0.7°. The reconstructions, performed using the NRecon software package (Skyscan), are based on the Feldkamp algorithm and resulted in axial grayscale images. The 2D images were created using CTAn software package (Skyscan) [50]. The 3D μCT models of each femur were created using a 3D reconstruction software package (Mimics 14.0, Materialise, Belgium) [50,51].

## Results

### Patient derived-prostate cancer bone metastasis tumor specimen generated tumors in immunodeficient mice

**Figure 1:**
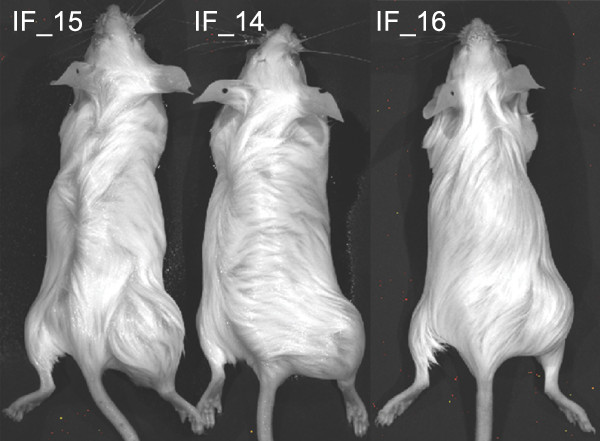
**Intra-femoral transplantation of PCSD1 (**P**rostate **C**ancer **S**an **D**iego 1) cells generated xenograft tumors in mice**. Tumor cells isolated directly from a patient-derived femoral bone metastasis were transplanted intra-femorally into Rag2^-/-^;γc^-/- ^male mice. Tumor growth was observed in the tumor-injected (right) leg of intra-femorally transplanted mice but not in the un-injected, contra-lateral (left) leg as shown in three representative mice 10 weeks post-transplantation.

A prostate cancer bone metastasis specimen was obtained from a castrate-resistant patient and transplanted sub-cutaneously or intra-femorally into immunodeficient, male *Rag2^-/-^;γ_c_^-/- ^*mice [52]. Minced tumor sample that was injected sub-cutaneously (SQ) produced xenograft tumors in all ten male *Rag2^-/-^;γ_c_^-/- ^*mice. Disaggregated primary tumor cells that were injected intra-femorally (IF) generated tumors in all eight *Rag2^-/-^;γ_c_^-/- ^*mice. As shown in Figure 1, tumors were evident in three representative mice at ten weeks in the tumor-injected (right) leg of all intra-femorally transplanted mice but not in the un-injected, contra-lateral (left) leg. Therefore, the take-rate of the primary tumor sample was 100% in both the sub-cutaneous and intra-femoral niches. Tumors harvested from both sub-cutaneous and intra-femoral tumors have been serially transplanted at least three times both sub-cutaneously and intra-femorally thus far: P0 (primagraft), P1 and P2. Low passage PCSD1 tumors were cryopreserved and serially passaged as intra-femoral and sub-cutaneous xenografts. Tumor take-rates are shown in Table 2. The lower take-rate in the intra-femorally injected mice is most likely due to the significantly fewer tumor cells injected into the femur than sub-cutaneously. Approximately 5,000 tumor cells were injected per femur which was ~10% of the total mixture of 50,000 cells injected IF per mouse. In contrast, the minced tumor pieces that were implanted sub-cutaneously were ~ 1 mm^3 ^containing approximately one million total cells. Mice injected IF with fewer as well as greater than 5,000 PCSD1 cells are currently being analyzed. Freshly harvested xenograft tumor cells as well as cryopreserved xenograft tumor cells have been used for long term *in vitro *culture experiments for testing novel compounds.

**Table 2:** PCSD1 tumor xenograft passaging and transplantation take-rate.

**Tumor Passage No**.	Number of mice injected	Number mice with tumor	Take-rate
**P0**			
SC	10	10	100%
IF	8	8	100%
**P1**			
SC	18	18	100%
IF	29	19	66%
**P2**			
SC	10	10	100%
IF	23	14	67%

Tumor passage number: P0 = primagraft: primary patient sample injected; P1 = first serial passage of tumor cells, that is, tumor cells harvested from P0 tumors are re-implanted into new mice and tumors allowed to develop; P2 = second serial passage of xenograft tumors; SC = sub-cutaneously transplanted tumors; IF = intra-femorally transplanted tumors.

### PCSD1 sub-cutaneous and intra-femoral xenograft tumors express PSA and AR

**Figure 2:**
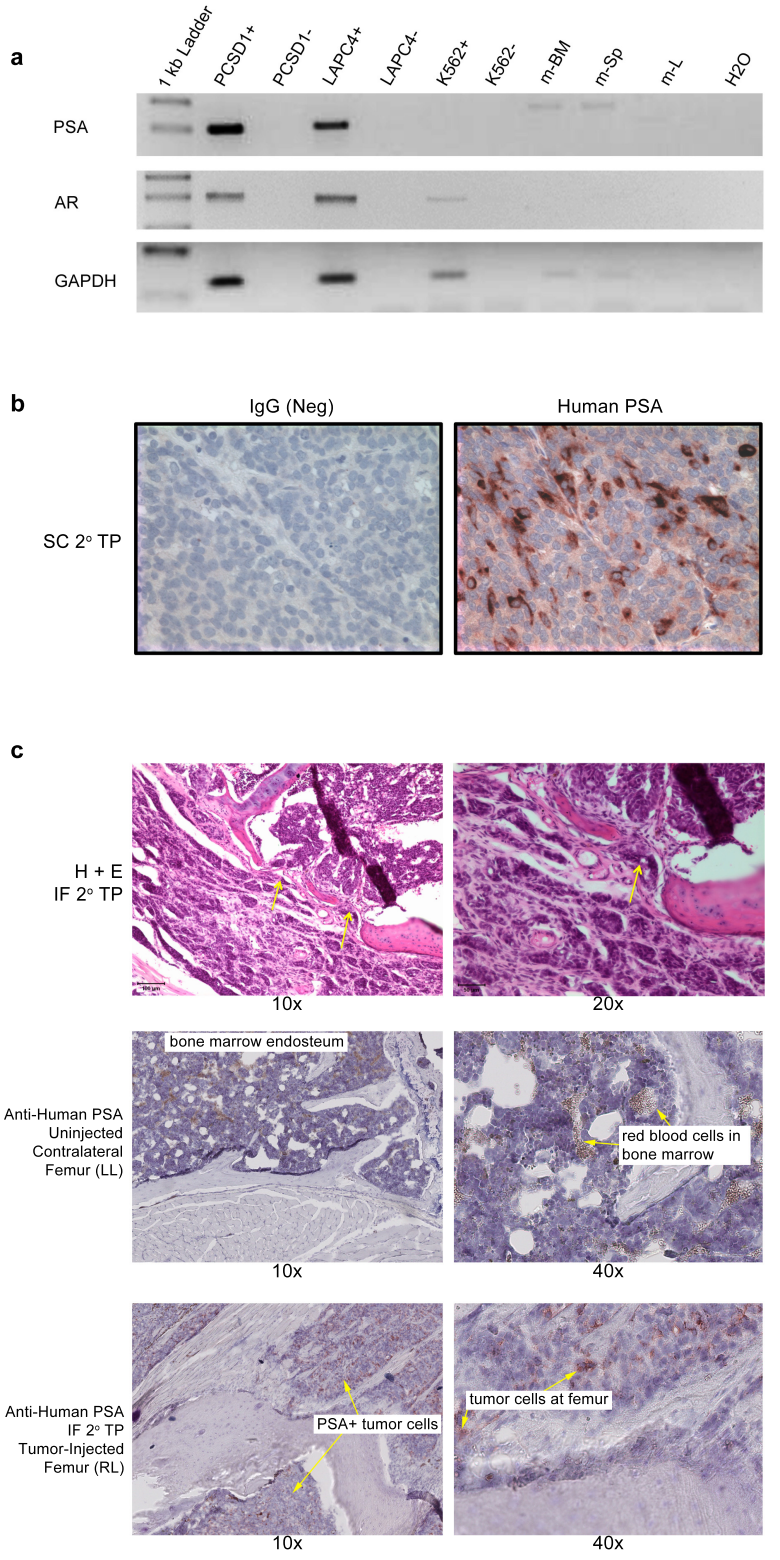
**PCSD1 xenograft tumors expressed human prostate PSA and human AR**. A. RNA was extracted from secondary transplant sub-cutaneous xenograft tumors (Passage 1, P1) and used for RT-PCR analysis of human prostate specific antigen (PSA) and human androgen receptor (AR). Human PSA and human AR-specific primers were used for PCR amplification of cDNA synthesized with reverse transcriptase (RT+) or without (RT-) and confirmed by sequencing of correctly sized bands. Human GAPDH-specific primers were used as an internal control. Human PSA and AR were expressed in the PCSD1 xenograft tumor and the human prostate cancer cell line, LAPC4, but not in the human chronic myelogenous leukemia (CML) cell line, K562. RNA from mouse spleen, bone marrow and liver did not express human PSA or AR. B. Immunohistochemical analysis showed human PSA protein expression in PCSD1 xenograft tumors. Images show paraffin embedded (PPFE) sub-cutaneous PCSD1 secondary transplant xenograft sections stained with IgG isotype negative control or human PSA-specific antibody. (C) Upper panels show H and E stained intra-femoral PCSD1 secondary transplant xenograft cryosections at 10× and 20× magnification. Middle panels show cryosections from the left, un-injected, contralateral femurs immunostained with human PSA-specific antibody at 10× and 40×. Arrows show red blood cells in bone marrow. Lower panels show cryosections from secondary intra-femoral transplants of PCSD1 immunostained with anti-PSA. Arrows point to human PSA positive (+) prostate cancer cells.

To demonstrate whether the xenograft tumors originated from prostate cancer in the patient bone metastasis specimen the expression of prostate specific antigen (PSA) was measured. Primers that were specific for the human gene target and spanned exon-intron boundaries were newly designed or selected from the literature as shown in Table 1 and the RT-PCR products verified by sequencing. As shown in Figure 2A, RT-PCR analysis showed the expression of human PSA in a sub-cutaneous PCSD1 xenograft tumor (P1) as well as in the human prostate cancer cell line, LAPC4, but not the human chronic myelogenous leukemia (CML) cell line, K562, nor murine bone marrow, spleen or liver [52]. Using primers for the full-length isoform of androgen receptor (AR) for RT-PCR demonstrated human androgen receptor (AR) expression in PCSD1 and LAPC4 (Figure 2A)[53,54]. Therefore, PCSD1 xenograft tumors originated from prostate cancer cells in the patient's femoral bone metastasis.

PSA protein expression was determined using immunohistochemical staining of PCSD1 xenograft tumor sections. Cytoplasmic PSA staining was detected in cells in sub-cutaneous PCSD1 xenografts (Figure 2B) and in intra-femoral xenografts from the right leg (Figure 2C, IF 2°TP (RL), lower panels). Cytoplasmic PSA staining was not observed in femoral sections from the un-injected, contra-lateral left leg (LL). Red blood cells in the bone marrow space that showed up as slightly reddish brown in color that was not due to PSA immunostaining were seen in the un-injected intra-femoral sections (Figure 2C middle panels). In the femur of the right leg, PCSD1 tumor cells were observed both in the endosteal bone marrow space where they were injected and having invaded extra-cortically surrounding the femur. Regions of osteolysis were observed in the immunostained sections and in H & E stained sections (Figure 2C, upper panels) through which the tumor may have invaded and migrated outside of the bone.

### PCSD1 xenograft tumors express luminal prostate biomarkers

**Figure 3:**
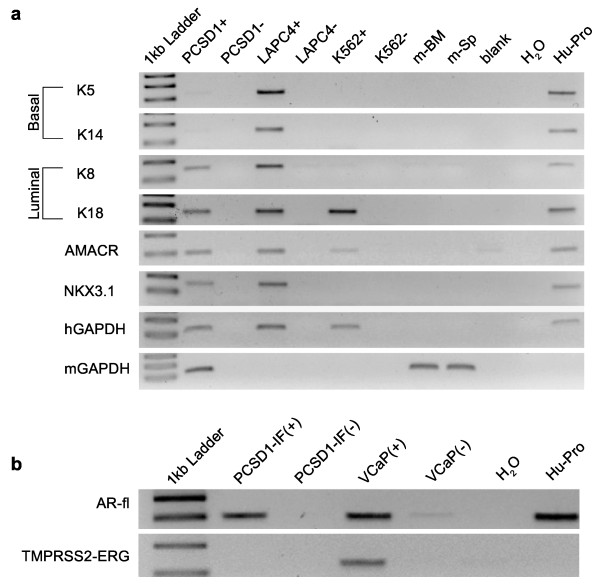
**PCSD1 xenograft tumors express luminal-type epithelial, advanced prostate cancer biomarkers**. A. RT-PCR analysis was performed on cDNA synthesized with reverse transcriptase (RT+) or without (RT-) from RNA purified from PCSD1 sub-cutaneous xenograft tumors, cultures of LAPC4, a human prostate cancer cell line and K562, a human CML cell line culture, as well as murine spleen, bone marrow and liver or H2O alone and RNA from normal human prostate tissue. Human specific primers for keratins 5, 8, 14 and 18, AMACR and NKX3.1, GAPDH and mouse-specific GAPDH were used to detect expression of these genes. B. RT-PCR analysis was performed on intra-femoral xenograft PCSD1 tumor cells, cultured VCaP prostate cancer cell line as well as normal human prostate tissue. Primers were specific for RNA from full length human AR or the TMPRSS2-ERG fusion gene. GAPDH levels were comparable.

Further molecular analysis to characterize PCSD1 tumors was performed using RT-PCR on additional human prostate biomarkers. Expression of keratins 5 (K5) and 14 (K14) are characteristic of basal prostate epithelial cells whereas keratins 8 (K8) and 18 (K18) are expressed in luminal prostate epithelial cells [44-46,55]. PCSD1 xenograft tumors expressed human K8 and K18 and very low levels of K5 and K14 (Figure 3A). Interestingly, LAPC4 expressed all four of the keratins. PCSD1 and LAPC4 both expressed the prostate transcription factor NKX3.1 [46]. PCSD1 and LAPC4 also expressed AMACR, a biomarker that is often up-regulated in advanced prostate cancer [56]. The human specific GAPDH and mouse specific GAPDH were expressed in the PCSD1 xenografts indicating the presence of both human and murine cells within the xenograft tumor [57,58]. Only human specific GAPDH was detected in the human cell lines LAPC4 and K562 that were grown in culture as expected. Correspondingly, the murine bone marrow and spleen tissues only expressed the mouse GAPDH. Taken together the results of the molecular analysis showed that PCSD1 is a luminal epithelial-type advanced prostate cancer [43-47].

The TMPRSS2-ERG fusion gene is a frequent genomic rearrangement in prostate cancers that results in placing the ERG ETS-family transcription factor under the androgen-regulated expression of the TMPRSS2 gene [59]. RT-PCR was performed to determine whether this gene fusion event was present in PCSD1. While the fusion transcript was detected in VCaP cells as shown previously [59], the TMPRSS2-ERG gene fusion was not detected in PCSD1 (Figure 3B). In addition, analysis of known alternative splicing variants of AR did not detect these in PCSD1 xenografts.

### PCSD1 intra-femoral xenograft forms mixed osteolytic and osteoblastic bone lesions

**Figure 4:**
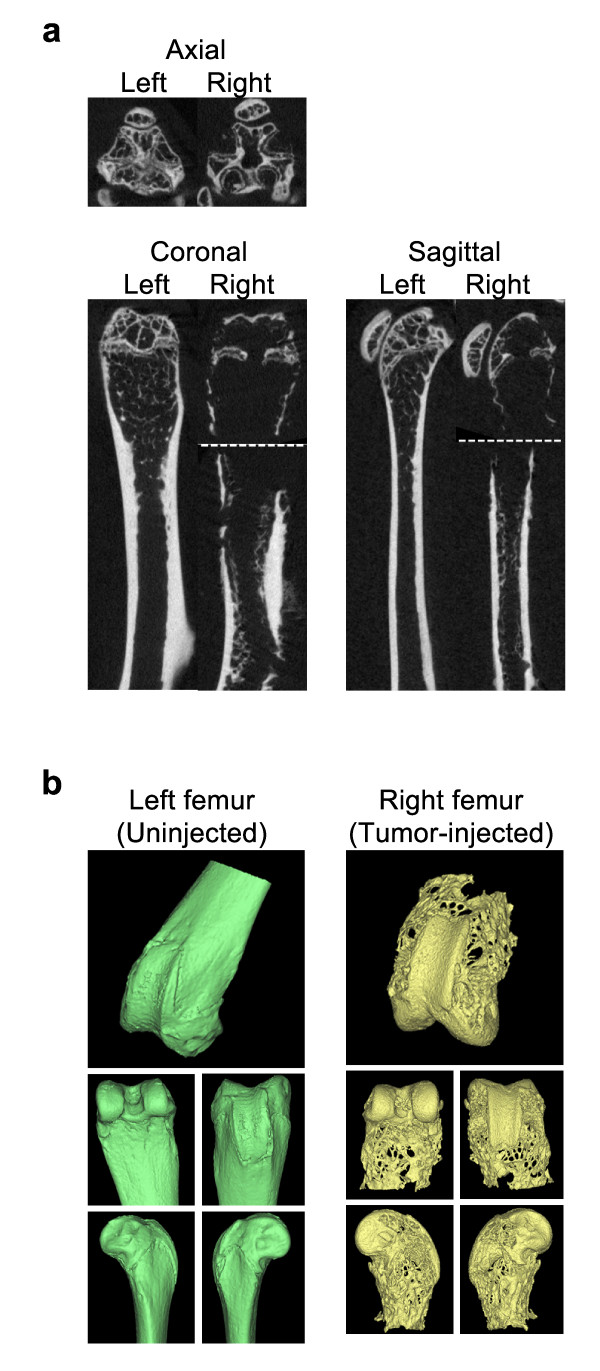
**MicroCT imaging of patient-derived intra-femoral (IF) PCSD1 xenografts revealed mixed osteolytic and osteoblastic bone lesions**. MicroCT scanning was performed on femurs and tibia isolated from mice injected with primary patient-derived tumor in the right femur showed that areas of increased bone density and sclerosis were apparent in the femur in which the tumor was growing as shown above for mouse IF15. A. P0 Bones osteolytic/osteoblastic microCT images. B. 3D reconstruction of distal femur.

**Figure 5:**
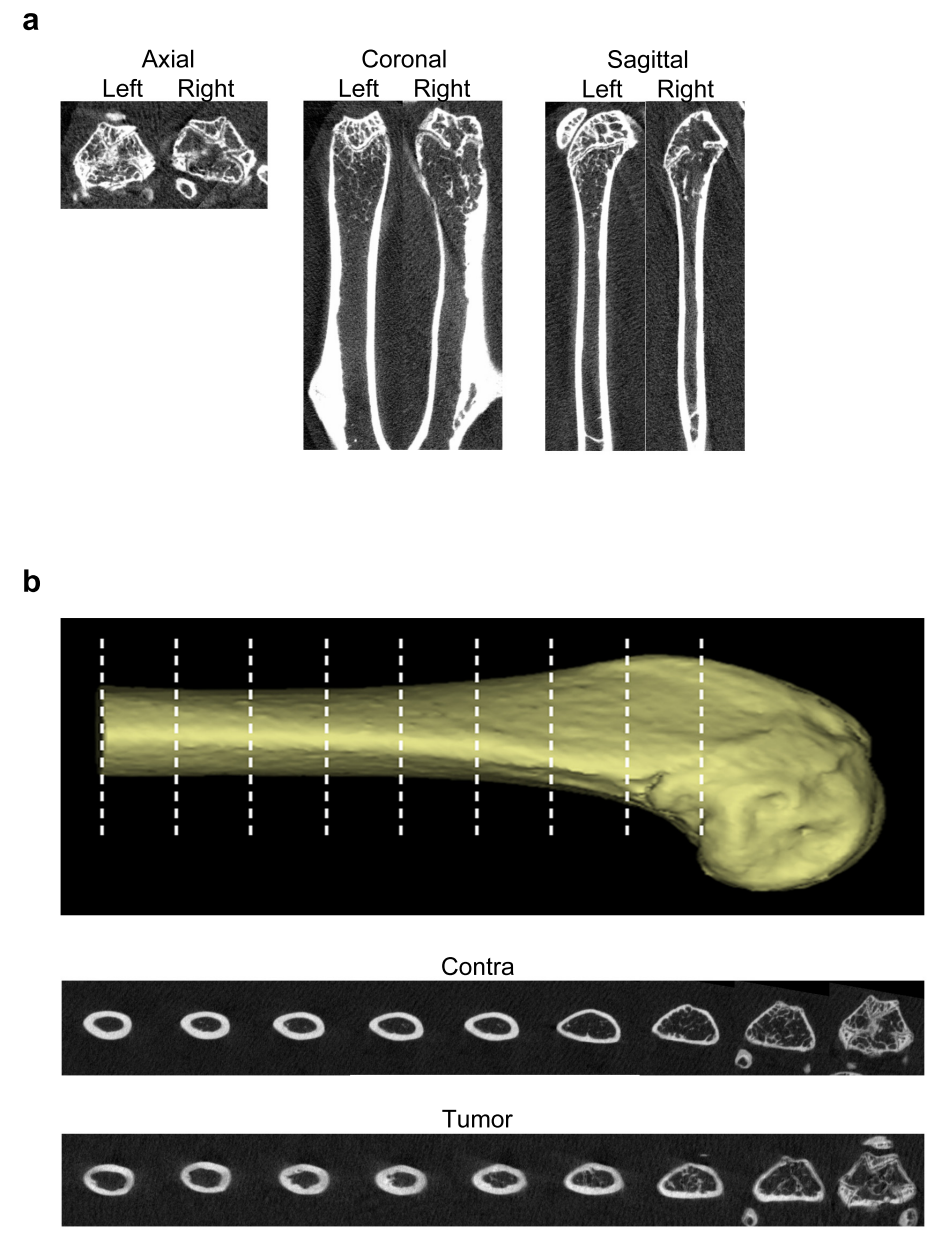
**MicroCT imaging of PCSD1 secondary intra-femoral transplanted xenograft showing osteolytic lesion at distal extremity of femur and osteoblastic lesion formation along shaft of femur**. A. Osteoblastic lesion MicroCT imaging of intra-femoral (IF) xenografts: microCT and cross-sections. B. Axial microCT scan series for comparison of femur cross-sections from un-injected, contra-lateral femur to PCSD1 tumor-injected femur.

Micro computed tomography small animal scanning (microCT) was performed on mice injected intra-femorally with PCSD1 to determine the effect of the growth of the tumors [31]. MicroCT scans from mice injected intra-femorally with the primary patient bone metastasis sample are shown in Figure 4. Regions with significant osteoporosis (bone thinning) as well as areas of bone sclerosis (increased bone density) were apparent in the femur in which the tumor was growing but not in the un-injected contra-lateral leg (Figure 4A). Three-dimensional (3D) reconstruction of the microCT scans revealed the extensively pitted, porous and eroded distal extremity of the femur from the tumor-injected (yellow) leg compared to the smooth femur surface contours of the contra-lateral, un-injected leg (Figure 4B). PCSD1 tumor growth produced significant osteolysis in the femur. In addition, regions of sclerosis or osteoblastic lesion formation were observed in the right, tumor injected femurs as shown in Figure 5A. MicroCT cross-sections along the length of the femur were compared to show the increased thickness and density of the femur in which PCSD1 tumor was growing compared to the un-injected femur (Figure 5B). The PCSD1 tumor induced bone lesion changes can be seen in the context of the whole animal in the movie of the 3D reconstruction of the microCT analysis (Additional file 1). The destructive effects of PCSD1 tumor growth on the internal and external surfaces of the distal femur are further shown in the 3D reconstruction microCT in Additional file 2. Therefore, *in vivo *microCT scanning revealed PCSD1 tumor growth produced mixed osteolytic and osteoblastic lesions. This recapitulated the mixed osteoblastic and osteolytic bone lesions observed in the patient.

## Discussion

Prostate cancer progression is marked by metastasis to bone, resistance to androgen deprivation therapy, radiotherapy and chemotherapy as well as the emergence of an apoptosis-resistant, tumor-initiating population for which there is no effective therapy [34,60-66]. There is a pressing need for new models to investigate prostate cancer interaction with the bone microenvironment and to develop therapies but they have been difficult to establish due to poor take-rates of xenograft transplantation of primary prostate tumors [17-19]. Here we describe PCSD1, a robust new patient bone metastasis-derived prostate cancer intra-femoral xenograft model for studying prostate metastatic bone disease. PCSD1 generated serially-transplantable sub-cutaneous and intra-femoral tumors when transplanted into immunodeficient *Rag2^-/-^;γ_c_^-/- ^*male mice. PCSD1 xenograft tumors were characterized as PSA^+^, AR^+^, K5^-^, K14^-^, K8^+^, K18^+^, AMACR^+^, NKX3.1^+^, and TMPRSS2:ERG^- ^human prostate cancer. These biomarkers identified PCSD1 as an advanced luminal prostate cancer bone metastatic cancer [43-47,56,63,64]. MicroCT analyses revealed PCSD1 formed mixed osteoblastic and osteolytic lesions in a murine femoral injection model which closely resembled the bone lesions in the patient [28,31,60].

The PCSD1 xenograft model will be used to understand the development of castrate-resistant prostate cancer in the bone microenvironment. Tumor growth of PCSD1 xenografts in intact versus surgically castrated mice is currently being measured. In culture, PCSD1 cells demonstrated androgen-independence as they can survive and proliferate without the addition on androgens.

Current standard-of-care therapies such as bisphosphonates, radiation, anti-androgens, chemotherapy, such as docetaxel, often eventually fail in patients who develop castrate-resistant prostate cancer [1,5,15,67-70]. The PCSD1 model will be used not only to elucidate mechanisms of failure of standard-of-care therapy but also to develop new therapies alone or in combination with current therapies.

The PCSD1 model will also be used to gain understanding of the unexpected, discordant effects of some new prostate cancer therapies that are being reported for bone metastatic prostate cancer. For example, in a Phase II Study of the new anti-androgen, Abiraterone, it was found that approximately one third of patients with chemotherapy-naive metastatic castration-resistant prostate cancer displayed bone scan flare discordant with PSA serologic response [71]. In other words, many patients with significantly lowered PSA levels after treatment with abiraterone still showed positive bone scans [71]. Conversely, some patients treated with the new c-Met tyrosine kinase inhibitor, Cabozantinib (c-Met TKI, XL184), showed dramatic reductions in positive bone scans but, paradoxically, no decrease in their PSA levels [72]. New bone metastasis models such as PCSD1 are, therefore, essential to understand the complex mechanisms of interaction of prostate cancer with the bone microenvironment and the variation in response to therapies in different patients, types of bone lesions or stages of bone metastatic prostate cancer progression.

## Conclusions

PCSD1 xenografts tumors were characterized as advanced, luminal epithelial prostate cancer from a bone metastasis using RT-PCR and immunohistochemical biomarker analyses. PCSD1 intra-femoral xenografts formed mixed osteoblastic/osteolytic lesions that closely resembled the bone lesions in the patient. PCSD1 is a new primary prostate cancer bone metastasis-derived xenograft model to study metastatic disease in the bone and to develop novel therapies for inhibiting prostate cancer growth in the bone-niche.

## Abbreviations

AR: androgen receptor; IHC: Immunohistochemistry; FACS: fluorescence activated cell scanning or sorting; IF: intra-femoral injection or transplantation; K562: Chronic myelogenous leukemia derived human cell line; LAPC4: Los Angeles Prostate Cancer cell line 4; LAPC9: Los Angeles Prostate Cancer cell line 9; Micro-CT: X-ray micro-computed tomography; P0: primagraft: primary patient sample injected; P1: first serial passage of tumor cells, that is; tumor cells harvested from P0 tumors are re-implanted into new mice and tumors allowed to develop; P2: second serial passage of xenograft tumors; PCSD1: Prostate Cancer San Diego 1 patient-derived xenograft or tumor cells; PSA: prostate specific antigen; *Rag2^-/-^;γ_c_^-/-^*: immunodeficient mouse strain with homozygous targeted deletions of Recombinase activated gene-2 and Interleukin 2 receptor common gamma chain; RT-PCR: Reverse transcription and polymerase chain reaction; SC: sub-cutaneous injection or transplantation; VCaP: vertebral metastasis of cancer of the prostate cell line.

## Competing interests

The authors declare that they have no competing interests.

## Authors' contributions

OR provided clinical expertise, selected, designed and performed RT-PCR and analyzed immunohistochemical images. AAK provided patient samples, clinical expertise in orthopedic oncology and interpretation of microCT scans. CW provided animal experiment expertise and performed intra-femoral injections assisted by HL. YBJ, performed RT-PCR and analyzed immunohistochemical images. KMS guided RT-PCR analyses and performed sequence confirmation. DG assisted with RT-PCR and primer design. TY and KM performed microCT scanning and analyses and generated 2D and 3D microCT images and movies; KM also performed scanning of intra-femoral PSA IHC slides. CHMJ provided expertise in generating primagraft and xenograft cancer models, bone marrow niche analysis as well as Rag2^-/-^;γc^-/- ^mice. SM provided clinical expertise and statistical analyses. NAC, provided molecular biology expertise and was involved in data analysis, biomarker selection and provided signal transduction expertise. CJK provided primary prostate tissue and tumor specimens, contributed to writing the manuscript and clinical expertise in prostate cancer. CAMJ is the PI of the study, wrote the manuscript, guided all aspects of generating the model and analysis. In addition, CAMJ performed patient sample preparation, xenograft tumor dissection and cell preparation for IF injections, RNA, DNA purification, tumor fixation, decalcification and mounting for sectioning and cryopreservation of tumor cells and sub-cutaneous injections. All authors have read and approved the final manuscript.

## Supplementary Material

Additional file 1 **Three-dimensional reconstruction of lower body microCT scan of mouse with intra-femoral PCSD1 xenograft at 10 weeks**. Effects of PCSD1 tumor growth in the right femur compared to the un-injected, contra-lateral, left femur including osteolysis in the right distal femur, periosteal reaction and "sunburst" appearance along the inner shaft of the right femur. Soft tissue mass of PCSD1 tumor can be seen around the right femur. Click here for file

Additional file 2 **Three-dimensional microCT reconstruction of the lower extremity of the right femur from a PCSD1 intra-femoral xenograft in a mouse showing exterior and interior surfaces of the bone lesion**. Click here for file
